# Percutaneous closure of postsurgical thoracic aorta pseudoaneurysms: the why, when, and how

**DOI:** 10.1007/s12471-023-01818-8

**Published:** 2023-09-04

**Authors:** Robbert J. de Winter

**Affiliations:** Department of Cardiology, Amsterdam University Medical Centers, Location Meibergdreef, Amsterdam, The Netherlands

Pseudoaneurysm of the aorta, disruption of the arterial wall with extravasation of blood contained by periarterial connective tissue and not by the arterial wall layers, can occur after dissection, trauma, endocarditis, in the setting of arteritis and after surgery. Postsurgical thoracic aorta pseudoaneurysm (PTAP) can arise from the cannulation site, clamping site, anastomosis site of venous grafts, valvulotomy site, or proximal or distal anastomosis after conduit placement. PTAP following cardiac surgery is rare, with its incidence reported to be 0.2–0.5% [1]. Approximately 50% of patients with PTAPs are asymptomatic [2], necessitating follow-up with a computed tomography (CT) scan in patients after surgery on the ascending aorta. The natural history of PTAP is basically unknown; historic series from the middle of the previous century report a 10-year survival rate of 30% in untreated patients, but these series included all types of aortic aneurysms (including 20% related to syphilis) [3]. Mortality associated with pseudoaneurysms after aortic surgery was reported to be as high as 61%; however, this was mortality related to abdominal aortic surgery [4]. Pseudoaneurysms are potentially fatal when they grow larger and rupture, or they can cause fistulas or compression of surrounding structures. Both European and American guidelines recommend treatment of aortic pseudoaneurysms [5, 6]. ESC guidelines state that ‘In patients with aortic pseudoaneurysms—if feasible and independently of size—interventional or open surgical interventions are always indicated’. Currently, no randomised studies are available that compare outcomes after open surgical and endovascular treatment in aortic pseudoaneurysm patients. The choice of treatment is commonly based on anatomical features, clinical presentation and comorbidities [5]. However, this relates to all pseudoaneurysms, both abdominal and thoracic. Thus, it seems reasonable to consider treatment of PTAP in all patients, weighing risks and feasibility of surgical or percutaneous intervention. Repeat surgery can be challenging and is associated with substantial morbidity and mortality [7]. Successful percutaneous treatment with an Amplatzer closure device was first reported in 2005 by Bashir et al. [8] and since then many case reports and small series have been published [9]. Depending on the size of the pseudoaneurysm, local anatomical factors, adjacent structures and operator preference and experience, atrial septal occluders, ventricular septal occluders, vascular plugs, duct occluders and coils have been shown to be successful [10].

In this issue of the *Netherlands Heart Journal*, Hegeman and colleagues report their experience with 11 cases of PTAP. The authors nicely illustrate the importance of three-dimensional imaging for procedure preparation and device selection and they include follow-up with CT scans [11]. They used different sizes of Amplatzer Vascular Plug III (AVP-III) and two atrial septal occluders. Importantly, they show that occlusion of the pseudoaneurysm was obtained in only four cases at short-term follow-up and significant residual flow remained in four cases on longer-term follow-up CT. Significant residual flow at long-term follow-up was present both with the AVP-III and septal occluder and was not predicted by residual flow on aortography at the end of the procedure. Patients will often take anticoagulants which cannot be discontinued, thus prohibiting thrombotic occlusion of the PTAP over time. Significant residual flow may predispose to endocarditis, especially when the original surgery was performed because of bacterial infection. Although repeat passage of a guiding catheter in the case of significant residual flow may be challenging, passage of a microcatheter and subsequent delivery of coils might have been an option.

Interestingly, there was no association between the size of the neck of the PTAP and the size of the device chosen by the operators. In addition, the choice of using the AVP-III in the majority of the 11 cases was not explained. Perhaps the oval shape of the device permitted avoiding obstruction of adjacent structures? The AVP-III is relatively soft and compliant compared to atrial septal occluders or patent foramen ovale occluders and therefore may reduce the risk of erosion and rupture of the PTAP? The aortic wall and implanted conduits are thick and relatively rigid and might be better suited for a less compliant device if adjacent structures are not a limiting factor. The study also demonstrates that repeat surgery in these patients at high risk of significant residual flow in the pseudoaneurysm after a percutaneous closure attempt is clearly not without risk.

In summary, this study adds to the growing body of evidence that percutaneous treatment of postsurgical pseudoaneurysms may be an attractive option when the risk of repeat surgery is deemed to be (too) high. Careful preparation with high-quality imaging is important; several options in terms of device selection, delivery guides and access routes are available. Long-term CT follow-up is essential to determine successful closure of the pseudoaneurysm.
